# Manualised Cognitive Remediation Therapy for adult obesity: study protocol for a randomised controlled trial

**DOI:** 10.1186/1745-6215-15-426

**Published:** 2014-11-04

**Authors:** Jayanthi Raman, Phillipa Hay, Evelyn Smith

**Affiliations:** School of Medicine, University of Western Sydney, Locked Bag 1747, Penrith, NSW 2751 Australia; Centre for Health Research, School of Medicine, University of Western Sydney, Locked Bag 1747, Penrith, NSW 2751 Australia; School of Medicine, James Cook University, Townsville, QLD Australia; Faculty of Health Sciences, University of Sydney, Lidcombe, NSW 2141 Australia; School of Psychiatry, University of New South Wales, Randwick, NSW 2031 Australia; Allied Health Research Unit, St Vincent’s Hospital, Darlinghurst, NSW 2010 Australia

**Keywords:** Cognitive Remediation Therapy, executive functioning, obesity, binge-eating

## Abstract

**Background:**

Research has shown that obese individuals have cognitive deficiencies in executive function, leading to poor planning and impulse control, and decision-making difficulties. An intervention that could help reduce these deficits and in turn help weight loss maintenance is Cognitive Remediation Therapy for Obesity (CRT-O). We aim to examine the efficacy of manualised CRT-O, which is intended to improve executive function, enhance reflective practice and help weight loss maintenance.

**Methods/Design:**

A randomised controlled trial (registered with the Australian New Zealand Clinical Trials Registry) will be conducted. First, 90 obese adults (body mass index >30 kg/m^2^) in the community will receive three weekly sessions of a group Behaviour Weight Loss Treatment (BWLT), and then will be randomised either to receive CRT-O or to enter a no-treatment control group. CRT-O training will comprise twice-weekly sessions of 45 minutes over a 4 to 6 week period, for a total of eight sessions. Measurement points will be at baseline, post CRT-O (or 4 to 6 weeks after BWLT for the no-treatment control), 3 months post treatment and 1 year post treatment. The primary outcome will be executive function and secondary outcome measures will include participants’ body mass index, hip to waist ratio, eating behaviours and quality of life.

**Discussion:**

This is the first study of its kind to examine the efficacy of Cognitive Remediation Therapy for obese adults through a randomised controlled trial.

**Trial Registration:**

Australian New Zealand Clinical Trials Registry number: 12613000537752. Date of registration: 14 May 2013.

## Background

Approximately 1 billion adults are currently overweight (body mass index (BMI) 25 to 29.9 kg/m^**2**^) and a further 475 million are obese (BMI >30 kg/m^2^) [1]. Ranking second only to smoking as a preventable cause of death, obesity contributes to over 300,000 deaths per year [2]. Obesity is also associated with a wide range of medical and psychological sequelae, eating disorder behaviours such as binge eating and subsequent impaired health-related quality of life (HRQoL) [3–5]. Although 5% to 10% weight loss is associated with clear health benefits, the prevention of weight regain has remained a challenge. Even with the help of professionals and extended behavioural treatments, weight regain typically occurs when professional contact ends [6]. By 3 to 5 years post-treatment, about 85% of patients have regained weight or even exceeded their pre-treatment weight [7].

Empirical research has found obesity to be associated with cognitive impairment, especially in executive function, irrespective of the presence of binge eating [8] and independent of co-morbid medical conditions such as diabetes, hypertension and cancer, which are themselves associated with adverse cognitive effects [9, 10]. Executive function encompasses a range of cognitive processes facilitating initiation, planning, regulation, inhibition, sequencing and achievement of complex goal-oriented behaviour [11]. The association of obesity and executive deficits is found across the age span, in children, adolescents, older adults, middle-aged adults and young adults [12–14]. A review [15] found that the evidence is highly consistent, with 22 out of 24 studies showing a negative association between obesity and cognition, especially in executive function. For example, inhibitory control, an aspect of executive function, involves the suppression of actions that are contextually inappropriate and interfere with behavioural goals. Studies of obese adults in the community have found executive deficits to be positively associated with disinhibited eating and greater food cravings [16]. Furthermore, obese individuals have been found to demonstrate cognitive impulsivity, an autonomic readiness to make quick decisions, which prevents successful inhibition of a prepotent behavioural response. It has been shown that impulsive subjects have marked limitations for learning suitable associations between reward and punishment [17, 18] and as a result they are more influenced by immediate rewards than by future consequence [18]. Hence, executive deficits have been shown to impair the capacity for impulse regulation, including control over the impulse to eat excessively [19]. Furthermore, evidence indicates a positive association between a higher BMI and decision-making impairments [20]. Findings from a study on the decision-making profiles of obese adult females using the Iowa Gambling Task showed a preference for high immediate reward, despite higher future losses in terms of both physical and psychological outcomes [18]. Studies have also shown that obese individuals perform similarly on the Iowa Gambling Task as those with anorexia nervosa, for example, see [21], and as patients with orbitofrontal dysfunction [22], and worse than those who are substance dependent [23]. While executive function deficits have been shown to be similar in anorexia nervosa and obesity, obese individuals show worse performance in the inhibition response measured by the Stroop test compared to individuals with anorexia nervosa or healthy controls [24].

The mechanism by which obesity is associated with cognitive function is, however, unclear at present [8]. Smith and colleagues [15] suggested a bidirectional relationship between obesity and executive function, where obesity impairs executive function via biological mechanisms, such as inflammatory markers or glucose abnormalities, and impaired executive function impacts on obesity by causing an inability to regulate food intake, plan ahead, inhibit responses or act impulsively. There is some evidence for this bidirectional association. One longitudinal study showed that poor executive function at age four predicted a high BMI at age six [14]. It is possible, based on the evidence presented above, that cognitive deficiencies may contribute to increased adiposity by exacerbating weight gain or regain after weight loss, and targeting these deficits in the obese has the potential to improve participants’ response to therapy. This led to the development of a novel treatment for weight disorders, namely Cognitive Remediation Therapy for Obesity (CRT-O) [15, 25].

CRT-O is a manualised treatment derived from Cognitive Remediation Therapy (CRT), which is used to treat people with addiction, feeding or eating disorders. In trials of people with a substance-use disorder, computer-assisted CRT has been shown to reduce individuals’ need for immediate reward and improve their decision-making [26]. Experimental studies have shown that practicing inhibitory control, an aspect of executive function, reduces the consumption of high-calorie foods [12, 27, 28]. Specifically, studies have shown that improving inhibition of behaviour towards high-calorie items, via a computerised go/no-go task, reduced the consumption of palatable foods across a 1-day period [12, 28]. Similarly, manualised Cognitive Remediation Therapy for Anorexia Nervosa (CRT-AN) has been shown to be associated with cognitive improvements in addition to improvements in eating-disorder-related quality of life at the end of treatment and eating-disorder psychopathology at a 6-month follow-up [29]. Furthermore, there were low dropout rates and high levels of acceptability among both patients and therapists [30].

Like CRT-AN, CRT-O is designed to provide individuals with the tools to think differently about their cognitive style and how they address food. However, in CRT-O the intent is to help maintain weight loss and prevent further weight gain by linking the thinking style to food intake and exercise behaviours. CRT-O is a brief eight-session individual therapy where simple cognitive tasks and exercises that encourage reflective learning and insight into a participant’s own thinking process are administered together with intra-session experimentation. The rationale being that this will improve various aspects of executive function, including cognitive flexibility, central coherence (attention to detail) and problem-solving and will in turn improve participants’ ability to follow the guidelines of a behavioural weight-loss programme. Supporting this are promising results from a small trial (*n* =44) by Verbeken *et al*. [12], testing executive function training with games in children. In this study, moderate to large effect sizes (standardised mean differences between 0.5 and 0.6) were found in executive function outcomes, such as improvements in a behaviour rating inventory of executive function and in a working memory task that favoured training. However, there were smaller effects for a task assessing response inhibition. To our knowledge the efficacy of this approach has not yet been investigated in adults.

We have designed the present randomised controlled trial (RCT) to test the efficacy of manualised individual face-to-face CRT-O as developed by Smith and colleagues [25]. The primary outcome in this superiority trial is improvement in executive function demonstrated at end of treatment and at 3 months, and maintained at the 1-year follow-up. Secondary outcomes are participants’ BMI, hip to waist ratio, eating behaviours and quality of life. Since studies on anorexia nervosa have found an improvement in quality of life, we wish to examine whether quality of life improves after CRT-O. In this RCT, all participants receive standard care for weight loss, namely group-based Behaviour Weight Loss Treatment (BWLT), and are then randomised to a CRT-O group or a no-treatment control group. CRT-O targets executive function deficits and changes in thinking style, whereas BWLT targets health literacy and weight-loss strategies including psycho-education, weight monitoring, dietary advice and exercise planning. Perceived barriers to weight management are discussed and challenged.

The primary hypothesis is that, compared to those who receive BWLT only, participants who receive BWLT plus CRT-O will demonstrate improved executive function post treatment, which is maintained at the 3-month and 1-year follow-ups. The secondary hypotheses are that that executive function changes will predict changes in weight, and compared to those who receive BWLT only, participants who receive BWLT plus CRT-O will have improved (i) weight loss (assessed by BMI and hip to waist ratio), (ii) HRQoL and (iii) dietary habits (including reduced binge eating) at post treatment and maintained at the 3-month and 1-year follow-ups.

## Methods/Design

### Participants

First, 90 obese individuals are recruited via direct advertisement to the community in Sydney, Australia. Inclusion criteria are: BMI ≥30 kg/m^2^, age 18 to 55 years, current weight under 180 kg, ability to provide informed consent and having completed 10 years of education in English. Participants who are depressed, have a binge eating disorder, are currently on psychotropic medication, have hypertension (medicated or not medicated), type 2 diabetes, or high cholesterol are eligible. Participants are excluded if they have a history of psychosis, head injury, neurological disorder including degenerative or inflammatory conditions or stroke, attention deficit hyperactivity disorder, epilepsy, developmental or intellectual disability; are unable to complete the testing (e.g. due to hearing, vision or language impediment); are on regular sedative or stimulant medication; and/or report regular substance use or abuse (for alcohol, more than two standard drinks five times a week). Individuals are also be excluded if they regularly use sedatives, hypnotics, antipsychotics, or anti-cholinergic or cholinergic medication. The sample size of 45 per group has been determined based on power estimates using Cohen’s tables for an estimated effect size of 0.6, power of 0.8, one-tailed test, *P* <0.05 and attrition of 20%. Although there are no previous studies of adults with obesity, these levels are commensurate with those achieved by Verbeken *et al*. [12].

### Procedure

**Figure 1:**
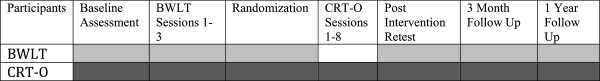
**Trial flow.** BWLT, Behavioural Weight Loss Therapy; CRT-O, Cognitive-Remediation Therapy for Obesity.

Recruitment was via advertisements placed on social media sites, university and community centre notice boards and via media interviews with journalists from metropolitan and community newspapers. The initial screen was by phone to establish potential eligibility. This was followed by a face-to-face psychological and neuropsychological assessment. All 90 participants then receive group-based BWLT that will run once a week for 3 weeks, each se**s**sion lasting 90 minutes. At the completion of the third and final session, participants are randomly allocated to CRT-O or no treatment. Randomisation and allocation concealment are conducted using an external computer-based program [31]. The RCT is registered with the Australian New Zealand Registry of Clinical Trials (trial id ACTRN12613000537752) (Figure 1).

### Assessment

After providing information on demographic and medical factors, including their weight, height, waist and hip circumferences, participants undergo a battery of neuropsychological and psychological tests. All assessment measures will be administered at baseline, after 7 to 9 weeks (3 weeks of BWLT and 4 to 6 weeks of either CRT or no treatment), and at the 3-month and 1-year post-CRT follow-ups. At the follow-ups, participants will also be asked whether they received any further treatment for their obesity. We will control for this in the final analyses.

#### Neuropsychological assessment

The six neuropsychological tests employed are selected because they cover different aspects of executive functioning and are commonly used in clinical and research settings. The tests are administered individually to all subjects by a trained clinical psychologist or neuropsychologist.

1. The Wisconsin Card Sorting Test (WCST) 64: The computerised version of WCST [32–34] will be used. In this test, subjects are instructed to categorise a series of cards according to one of three stimulus features (colour, shape or number of illustrations on them), without this principle being revealed to the subject. After each association, the only feedback given is whether a match is correct or incorrect, the idea being that subjects should infer how to categorise based on the feedback they receive. This test measures categorisation, inference, testing of hypotheses, cognitive flexibility, cognitive inhibition and response to feedback [16]. The number of total errors and perseverative errors and failure to maintain a set are the dependent measures. WCST is considered to be a valid measure of abstract reasoning ability to maintain an appropriate planning and problem-solving strategy across changing stimulus conditions to achieve a future goal, and it is the most widely used test of executive function [35].
2. Digit Span, a subtest of the Wechsler Adult Intelligence Test III [36]: In this test, the subject is presented with a series of digits to be repeated in the same order (Digit Span Forward) followed by another series to be repeated backwards (Digit Span Backward). Digit Span Forward assesses the phonological loop and Digit Span Backward assesses central executive aspects of the verbal working memory [37]. The score is calculated based on the number of correct responses that could be immediately retained.
3. Trail Making Test (TMT) [38]: This will be used to assess psychomotor speed, visual integration, cognitive flexibility and inhibitory control. This test measures the subject's ability to connect written numbers in an ascending order (Trail A), and, afterwards, to connect numbers and letters, alternating numbers in ascending order and letters in alphabetical order (Trail B), for example, 1-A-2-B. The score is the response time taken to complete the respective trails [35].
4. Computerised Reward–Loss Task [39]: This is a probabilistic classification task that assesses decision-making. In each trial, participants view one of four images and are asked to guess whether it belongs to category A or B. In the reward-learning task, if the participant correctly guesses the category membership for a trial, a reward of +25 points is received. If the participant guesses incorrectly, no feedback is given. In the punishment-learning task, if the participant guesses incorrectly for a trial, a punishment of -25 points is received, and correct guesses receive no feedback. The reward and punishment trials are intermixed so that no-feedback trials are ambiguous. The ±25 points symbolise quarter dollars that are won or lost, but no real payments are used in this study. The dependent variables are the number of errors made and completion time.
5. Behaviour Rating Inventory of Executive Function, Adult Version (BRIEF-A) [40]: This is a widely used, standardised measure based on a multidimensional model of executive function and the only available self-report measure of executive function at present. The BRIEF-A captures information on an adult’s executive functions or self-regulation in his or her everyday environment.
6. Rey–Osterrieth Complex Figure Test [41, 42]: This test (both copy and a 3-minute recall) will be used to measure attention to detail (central coherence), perceptual and organisational skills, and nonverbal memory. Scores will be calculated for the copy and 3-minute delay portions of the test using the scoring manual.

#### Pre-morbid IQ

The Australian version of the National Adult Reading Test, Revised [43, 44] is administered to control for pre-morbid intelligence. This is a widely used reading test that provides an estimate of pre-morbid IQ. It consists of 50 irregularly spelled words, listed roughly in order of difficulty. The test involves participants being presented with a word card and instructed to read each word out aloud. Interviewers then record correct pronunciations, with the total number of correct responses being the score.

#### Anthropometric measures

For participants, we measure: (1) height and weight with calibrated scales (from which we will derive BMI in kg/m^2^), and (2) hip and waist circumferences to calculate the waist to hip ratio.

#### Demographics and medical history

Age, gender, education and socio-economic status (by postcode) are recorded in the general questionnaire. This form also includes medical history, current medical conditions and medication.

#### Nutrition and lifestyle behaviours

These are assessed using a written 24-hour dietary recall method [45]. A brief questionnaire examining basic food group frequencies and portion sizes, and a questionnaire on frequency and intensity of exercise are used, both adapted from large Australian population questionnaires in the Australian Women’s Health project [46] and the National Nutrition Survey, Australia [47].

### Psychological assessment

The Clinical Obesity Maintenance Model [48] proposed by Raman and colleagues argues that psychological variables, such as habitual cluster behaviours, emotional dysregulation and mood, interact with executive functioning and impact on the overeating and binge eating behaviours of obese individuals. Hence, the following measures are included in this RCT to investigate the broader range of maintaining mechanisms, including but not limited to executive deficits, and to ensure both groups are equivalent at baseline in other measures that may impact on weight.

#### Depression, Anxiety and Stress Scales

Depression symptoms in the obese in this study are measured by the 21-item Depression, Anxiety and Stress Scales [49]. Obesity has been shown to increase the risk of developing depression and depression has been found to be predictive of obesity [50], and there is a dose–response gradient in that the association between depression and obesity was stronger than the association between depression and being overweight [51]. There is ample evidence that depressive disorders are also associated with deficits in attentional functions, executive control and lowered cognitive flexibility [52]; verbal learning and memory [53] and interpretation biases [54–57]. This test has been validated in a number of clinical and non-clinical populations and is psychometrically sound with good reliability and validity to measure depressive symptoms [49, 58].

#### Eating disorder symptoms

Individuals with eating disorder symptoms, such as binge eating, may have more difficulty losing weight [59] and hence this will be controlled for in our analyses. Eating disorder symptoms are measured by the Eating Disorder Examination Questionnaire [60]. Acceptable internal consistency and test–retest reliability have been demonstrated for this questionnaire [61]. It has 36 items and measures concerns about shape, weight and eating; restraint and self-reported binge eating. Subscale scores for shape, weight and eating concerns and restraint range between 0 and 6. A higher score indicates more severe eating psychopathology.

#### The Grazing Questionnaire

The Grazing Questionnaire [62] is used to measure the participants’ food grazing behaviours, i.e., to assess whether there is a pattern of continual overeating. Grazing is increasingly recognised as an important eating behaviour associated with obesity [62]. This questionnaire has been validated in a university sample of both genders and has demonstrated high internal consistency, test–retest reliability and convergent validity at initial testing [62].

#### Habit clusters

Habit clusters are measured by the Modified Self-Reported Habit Index [63]. Research has shown that when cognitive resources become limited, individuals are inclined to make heuristic-based choices [64]. This is reflected in obesity-maintaining dietary habits. Given the habitual nature of eating and the rapidity with which people make eating decisions [64], the behaviours are likely to be the consequence of automatic responses to contextual food cues, many of which lead to increased caloric consumption and poor dietary choices [65, 66]. This index has been shown to be a valid and reliable scale to measure physical activity habits [67]. For the purposes of this study, this index has been modified to include other obesity-related behaviours such as sedentary behaviours, smoking and alcohol consumption.

#### Emotion dysregulation

Emotion dysregulation is measured by the Difficulties in Emotion Regulation Scale (DERS) [68]. Emotion regulation has been described as a continuous, dynamic system responsive to all emotional experience, consisting of both autonomic and controlled processes [69, 70], perhaps mediated by executive function [48]. Since it relates to obesity, problems in attentional and inhibitory control have been associated with binge eating and eating pathology in adults [71]. The DERS is a 36-item self-report questionnaire that assesses clinically relevant difficulties in emotion regulation (with a particular emphasis on negative emotions). There is evidence to support the reliability of DERS scores. Specifically, DERS scores have been found to demonstrate good test–retest reliability [72] and the overall DERS score and subscale scores have been found to have high internal consistency within both clinical, for example [68, 73], and nonclinical populations, for example [68, 74]. Support for the construct and predictive validity of DERS scores within both clinical and nonclinical populations has also been found, for example [69].

#### Health-related quality of life

HRQoL is measured with the 12-item Short Form [75], which is a widely used quality of life measure with good construct and criterion validity and with adequate sensitivity to change.

### Behavioural weight-loss group intervention

The BWLT in this RCT targets diet and exercise through behavioural modification techniques over three 90-minute weekly sessions. In the first session, obesity-related risk awareness, challenges with perceived barriers and perceived personal control are discussed in depth. Cognitive challenging techniques are taught and in-session goal setting practices are conducted. In the second session, participants receive comprehensive education about nutrition, hunger management and healthy eating practices. Problem-solving techniques are taught so that participants can effectively deal with difficult situations that threaten their weight-control efforts. National physical activity guidelines are discussed and further training given on goal setting and goal achievement. In this last session, attention is given to motivation enhancement and relapse prevention strategies to help individuals maintain their weight loss.

### Cognitive Remediation Therapy for Obesity

CRT-O is a manualised intervention that consists of mental exercises aimed at improving cognitive strategies, thinking skills and information processing through practice. CRT, in particular, promotes reflection on thinking styles, develops metacognition and helps to explore and apply new thinking strategies in everyday life. The primary function of CRT is to improve the thinking process rather than the content [76–78]. In the absence of a published manual, a CRT-O manual was developed to meet the needs of the obese participants for the purposes of this RCT. The intervention is conducted face-to-face and delivered by the first author (JR), who is a registered clinical psychologist in Australia. The principles, structure and main components of the original CRT-AN are maintained. Some important modifications, however, have been made to adapt the manual for obesity and these are described in Smith *et al*. [25].

### Treatment fidelity

The first author (JR) was trained in a workshop on CRT-AN by the developers of this treatment approach (Kate Tchanturia and Helen Davies, Institute of Psychiatry, King’s College London, UK). The third author (ES) piloted the different versions of manualised CRT-O with three people. Concomitant interventions outside of the trial setting are neither encouraged nor prohibited during the intervention phase. To increase transparency and documentation as well as for quality and treatment fidelity assessment, 10% of the sessions will be audiotaped.

### Data collection, management and analysis

Data is collected at baseline, end-of-treatment and the 3-month follow-up by the trial therapist. An independent assessment, blind to intervention group, will be made at the 1-year follow-up. Data is entered, cleaned and coded by a research assistant, blind to group allocation, and stored at a secure university facility according to university protocols. An independent statistician will conduct all preliminary analyses. The Statistical Package for Social Sciences (SPSS) statistics will be used.

Neuropsychological measures will first be examined independently and will then be integrated in an executive function composite following the procedure recommended by Rosnow and Rosenthal [79, 80] and applied by Clark *et al*. [81]. The composite score will be generated by converting each executive function scale across all assessment points to *z*- scores, and then averaging across the measures.

Descriptive statistics will be used to present demographic and clinical data including eating disorder features. Baseline univariate between-group tests will be used to compare outcome variables and clinical and demographic data for the groups. Data will be analysed following the intention-to-treat principle. Linear mixed effects modelling [82] will be used to test for between-group differences in the continuous outcome measures, namely the composite scores of cognitive function, waist circumference and BMI, and potential confounds (e.g., levels of depression, anxiety, stress and emotion dysregulation). Generalised estimating equations [83] with a logit response function, or logistic regression, will be used for dichotomous outcomes, such as achieving a 5% reduction in body weight. Effect sizes between and within the two groups will be calculated with Cohen’s *d* computed with the pooled standard deviation, and the odds ratio will be presented where appropriate.

### Ethics and dissemination

The study has been approved by the Human Research Ethics Committee of the University of Western Sydney (H9787). Written informed consent has been obtained from each subject after thorough information about the study was provided. Participants were advised that all data collected will be de-identified prior to analysis and stored securely in electronic and paper forms. All identifying information (e.g., signed consent forms) will be kept separate from other data and will be held in confidence. Only investigators and authorised research personnel will have access to the data. The results will be submitted to peer-reviewed scientific publications, presented at national and international scientific meetings, presented to relevant community fora and released in lay synopsis form to media outlets.

## Discussion

Obesity is an important global public health issue. Due to the long-term failure of interventions for obesity, there is a desperate need to develop novel and innovative approaches to treatment. In particular, weight-loss maintenance remains a major challenge. Obese individuals are known to suffer from significant cognitive deficits, especially in executive function. We have identified the possibility that an intervention such as CRT could improve treatment outcomes and has the potential to improve current therapy practices.

The application to CRT for obesity is highly novel. Executive function deficiencies in the obese are a potential target and unlike other contributing factors (e.g. diet and exercise) have not previously been investigated. CRT-O, which aims to improve specific deficiencies such as inflexibility, poor problem-solving and poor attention to detail (central coherence), could have benefits for the health of the increasing number of obese individuals in our community and reduce the economic burden from obesity and its medical and psychological sequelae. Our study is designed to compare two groups, a BWLT and CRT-O group with a BWLT and no-treatment group, to allow us to draw conclusions about the specific efficacy of CRT-O following BWLT training compared to no treatment.

The strengths of our study design include adequate concealment of randomised group allocation, use of validated and standardised assessments, and independent blinded data analysis. A limitation is that follow-up assessments are blinded only at 1 year. However, this is an important outcome time, since to be clinically meaningful for longer-term weight-loss maintenance, improvements in cognition need to be sustained over time. Furthermore, the Institute of Medicine has defined criteria for assessing successful weight maintenance as a weight loss of ≥5% of body weight that is maintained for at least a year [84]. For the purposes of this study, the Institute of Medicine’s criteria will be used as a standard definition of successful weight maintenance at this study's 1-year follow-up. Another limitation is that therapeutic time with a therapist is not matched between conditions and participants are not blind to condition, i.e., we are not employing a control–therapy comparison. As obesity tends to get worse without treatment, at our 1-year follow-up, we will examine whether individuals’ BMI is significantly higher in the no-treatment group compared to the CRT-O group. We will also control for any additional treatment each group received in that time and whether the change of executive function predicted that weight loss.

The design of this RCT offers a pathway to address, for the first time, the integral aspects of executive function and its impact on obesity, and their interaction with other maintaining factors such as emotion dysregulation, depression and habitual cluster behaviours. It is hoped that the findings of this RCT will provide an important first step towards an understanding of the potential of CRT in obesity. Based on the limited success of current behavioural weight-loss programmes and coupled with the data on the executive function deficiencies in obesity, it appears likely that improving an individual’s executive function has the potential to enhance treatment outcomes for obesity.

## Trial status

Recruitment is now complete; therapy and data collection are in progress.

## Abbreviations

BMI: body mass index
BRIEF-A: Behavior Rating Inventory of Executive Function, Adult Version
BWLT: Behaviour Weight Loss Treatment
CRT: Cognitive Remediation Therapy
CRT-AN: Cognitive Remediation Therapy for Anorexia Nervosa
CRT-O: Cognitive Remediation Therapy for Obesity
DERS: Difficulties in Emotion Regulation Scale
HRQoL: health-related quality of life
RCT: randomised controlled trial
WCST: Wisconsin Card Sorting Test.
